# Genetic analysis of infectious bronchitis virus (IBV) in vaccinated poultry populations over a period of 10 years

**DOI:** 10.1080/03079457.2023.2177140

**Published:** 2023-02-24

**Authors:** Cornelis J. Vermeulen, Remco Dijkman, J. J. (Sjaak) de Wit, Berend-Jan Bosch, J. A. P. (Hans) Heesterbeek, Gerdien van Schaik

**Affiliations:** a Department of Population Health Sciences, Faculty of Veterinary Medicine, Utrecht University, Utrecht, The Netherlands; b Royal GD (GD Animal Health), Deventer, The Netherlands; c Virology Division, Department of Biomolecular Health Sciences, Infectious Diseases & Immunology, Faculty of Veterinary Medicine, Utrecht University, Utrecht, The Netherlands

**Keywords:** Infectious bronchitis virus, poultry, vaccination, spike protein, sequence analysis, selection, N-glycosylation, divergence

## Abstract

Infectious bronchitis virus (IBV) is an avian pathogen from the Coronavirus family causing major health issues in poultry flocks worldwide. Because of its negative impact on health, performance, and bird welfare, commercial poultry are routinely vaccinated by administering live attenuated virus. However, field strains are capable of rapid adaptation and may evade vaccine-induced immunity. We set out to describe dynamics within and between lineages and assess potential escape from vaccine-induced immunity. We investigated a large nucleotide sequence database of over 1700 partial sequences of the S1 spike protein gene collected from clinical samples of Dutch chickens submitted to the laboratory of Royal GD between 2011 and 2020. Relative frequencies of the two major lineages GI-13 (793B) and GI-19 (QX) did not change in the investigated period, but we found a succession of distinct GI-19 sublineages. Analysis of dN/dS ratio over all sequences demonstrated episodic diversifying selection acting on multiple sites, some of which overlap predicted N-glycosylation motifs. We assessed several measures that would indicate divergence from vaccine strains, both in the overall database and in the two major lineages. However, the frequency of vaccine-homologous lineages did not decrease, no increase in genetic variation with time was detected, and the sequences did not grow more divergent from vaccine sequences in the examined time window. Concluding, our results show sublineage turnover within the GI-19 lineage and we demonstrate episodic diversifying selection acting on the partial sequence, but we cannot confirm nor rule out escape from vaccine-induced immunity.

**RESEARCH HIGHLIGHTS**
- Succession of GI-19 IBV variants in broiler populations.
- IBV lineages overrepresented in either broiler, or layer production chickens.
- Ongoing episodic selection at the IBV S1 spike protein gene sequence.
- Several positively selected codons coincident with N-glycosylation motifs.

## Introduction

Infectious bronchitis (IB) in chicken is caused by the *Gammacoronavirus* infectious bronchitis virus (IBV) that causes major health issues which result in significant economic losses. To protect their flocks, many poultry farms have adopted vaccination programmes with one, or a combination of live attenuated and inactivated vaccines which are routinely applied. A successful vaccination is able to greatly reduce the IBV transmission among vaccinated chickens (de Wit *et al*., 1998). IBV has circulated for decades in populations of livestock with a long history of massive routine vaccination of chickens.

RNA viruses like IBV can rapidly adapt to evade natural, or vaccine-induced immunity (Drummond *et al*., 2003). For instance, it has been demonstrated that within the time span of two decades human coronavirus 229E has diverged sufficiently from ancestral sequences to evade antibody immunity (Eguia *et al*., 2021). In the recent pandemic, the succession of SARS-CoV-2 variants also demonstrated the capacity of the virus to adapt to evade immune responses (Lazarevic *et al*., 2021). In a similar way, vaccine-induced immunity is expected to drive antigenic evolution in IBV. Supporting evidence for adaptive evolution of IBV in response to the introduction of homologous vaccines has been described by Franzo *et al*. (2019). Evolution of IBV has recently been reviewed by Legnardi *et al*. (2020).

We have collected IBV sequence data from clinical samples from chickens spanning a time period of 10 years. The data set consists of ∼350 nucleotide-long fragments of the gene coding for the S1 subunit of the spike protein. This protein is known to evolve rapidly since the most relevant epitopes are located here. The fragment spans one of three hypervariable regions (HVR3) in the gene providing high resolution to distinguish circulating lineages. The HVRs are known to be associated with epitopes for neutralization (Koch *et al*., 1990; Cavanagh *et al*., 1992; Moore *et al*., 1997; Ambepitiya Wickramasinghe *et al*., 2014). The immune response is believed to be the main selective force acting on the antigenic regions of IBV genomes (Legnardi *et al*., 2020). In addition, the spike glycoprotein is the major determinant of infectivity. The glycans coating the spike protein are known to serve roles in protein folding, viral receptor binding and viral entry and to shield the spike protein from neutralizing antibodies (Wei *et al*., 2010; Zheng *et al*., 2018).

In this study, we examined the evolution of IBV with the aim to describe within and between lineage dynamics and explore several factors that may effect this. As immune evasion is an obvious candidate driving spike protein evolution, we investigated whether we could detect the divergence of sequences from known vaccine sequences. To explore the functional consequences of sequence evolution, we assessed the distribution of N-linked glycosylation sites.

## Materials and methods

### Viral sequences

The initial collection of sequence data included 2077 sequences of a ∼350 nucleotide fragment of the S1 gene of IBV. This fragment covers residues 241–352 in the C-terminal domain of the translated sequence of the S-gene of the Beaudette reference sequence (GenBank accession M95169.1) and overlaps hypervariable region 3 (HVR3). The respiratory and cloacal samples were derived from IBV-suspected Dutch chicken samples submitted to Royal GD between 2011 and 2020. Samples from both broiler and layer production were included. Approximately half of the samples were obtained from birds that were submitted for *post mortem* examination and half were from sample swabs sent in for PCR-based diagnostic tests. Submission is voluntary and based on clinical suspicion of IB in the flock by the farmer, or veterinarian. All samples that tested positive for IBV were being sequenced. Samples from countries other than the Netherlands were excluded from the analyses.

### RT–PCR and partial S1 sequencing

For routine testing, viral RNA was extracted using the High Pure Viral Nucleic Acid kit (Roche Applied Science, Penzberg, Germany) according to the manufacturer’s instructions. Two RT–PCR primer sets were used, a universal IBV RT–PCR generating a fragment of approximately 350 base pairs of the S1 gene with primers XCE1 + and XCE3- (Cavanagh *et al*., 1999) and a genotype-specific RT–PCR for D1466-like strains using forward primer 5′-TACRggMAATTTTACTgATgg-3′ and reverse primer 5′-CTgACTgCTTACAAgAACC-3′. Both RT-PCRs were performed using a VeritiTM Thermal Cycler (Thermo Fisher Scientific, Waltham, MA, USA) in combination with the Qiagen one-step RT–PCR kit (Qiagen, Hilden, Germany) under the following conditions: reverse transcription reaction for 30 min at 50°C; denaturation 15 min at 95°C, followed by 45 cycles with 30 s at 95°C, 30 s at 50°C and 60 s at 72°C. The S1 amplicons were separated on a 1% agarose gel, and visualized with ethidium bromide staining and an ultraviolet light transilluminator. The purified amplicons were sequenced bidirectionally using Sanger sequencing with both the XCE1+ and XCE3-primers (BaseClear, Leiden, the Netherlands). Consensus sequences were constructed using MEGA7.0 (Kumar *et al*., 2016).

### Reference panel and typing

IBV strains were typed by comparison against an internal database of 97 reference sequences. The subset of sequences identified in our final data set is provided in the Online Supplementary Table 1. Viral sequences were typed by finding the closest match in the reference panel at a minimum of 95% nucleotide sequence identity. Identity was defined as the number of identical nucleotides divided by the total number of nucleotide positions in the alignment of both sequences.

### QC procedure

We performed quality control on the initial collection by enforcing the following criteria. We removed sequences with total lengths that were not divisible by three indicating frameshift mutations. We removed sequences with internal stop codons and all degenerate consensus sequences. Outlier sequences, as determined by visual inspection using principal component analysis (PCA) performed in PLINK 1.90 (Chang *et al*., 2015) were removed as well. Since it is known that recombinant sequences complicate phylogenetic analysis (Valastro *et al*., 2016) we removed 26 sequences typed as Xindadi-like strains being recombinants of GI-13 and GI-19 lineages (de Wit *et al*., 2021). Finally, we found untyped sequences that were nested within well-typed clades in a phylogenetic tree. Since close examination revealed those sequences to be from mixtures, we removed those as well. The numbers of rejected sequences at each step are shown in Online Supplementary Figure S1.

### Multiple sequence alignment and phylogenetics

Multiple sequence alignment was performed on translated sequences by MUSCLE 3.8.31 using default settings (Edgar, 2004). Initial trees for QC-purposes were built from the dataset using FastTree2 Double Precision version 2.1.11 under the general time reversible (GTR) model with discrete gamma-distributed site-rate heterogeneity (Price *et al*., 2010). Prior to tree building, we removed duplicate sequences. For the final tree we used IQ-TREE multicore version 2.2.0-beta with the -m GTR flag (Nguyen *et al*., 2015). The GI-13 and GI-19 lineages were identified by collecting all sequences descended from the MRCA of sequences typed as QX-D388 (de Wit, Nieuwenhuisen-van Wilgen, *et al*., 2011) for GI-19 and either 4/91, CR88, or 1/96 for GI-13, respectively.

### Episodic selection

In order to detect occurrences of episodic positive selection acting on individual codons, the sequence alignment was submitted to the mixed effects model of evolution (MEME) as implemented in the HyPhy package (Murrell *et al*., 2012). Among the various methods offered as part of the HyPhy package, MEME is the recommended method for detecting positive selection acting at individual sites. This is the appropriate method for our data set where clade-specific episodic selection on a few selected sites may be operating. MEME uses the ratio of the nonsynonymous substitution rate (*β*) to the synonymous substitution rate (*α*), expressed as *ω*, to detect selection allowing the value of *ω* to vary among different sites and different branches. The output of MEME contains two categories of estimates per codon site: the proportion of branches *p^-^* evolving under selection with the strength of selection *ω^-^* and the remaining proportion of branches *p^+^* evolving with the strength of selection *ω^+^*. The estimate *ω^-^* is constrained to be no higher than 1 and serves as an estimate of the strength of purifying selection. The estimate *ω^+^* is not restricted and is taken to signify positive selection operating at a given site when its value is significantly higher than 1. Note that when positive selection is operating on a subset of branches, it is not pervasive but referred to as episodic selection. Since high non-synomous substitution rates act to maintain amino-acid diversity, significantly evolving sites are taken to be under diversifying selection as opposed to directional selection. HyPhy MEME was run with default settings and we accepted the default significance threshold of 0.1 to signify positive selection acting at a site. We controlled the false discovery rate at 10% by applying Benjamini-Hochberg correction on the overall *P*-values reported per site by HyPhy MEME (Benjamini & Hochberg, 1995).

### Genetic variation

Prior to estimation of genetic variation, 62 divergent sequences were removed, as collection effort for divergent sequences was increased after 2016. We quantified nucleotide diversity *π* by using:

$$\pi =\frac{2S}{\left(n\cdot (n-1)\cdot L\right)}$$

Where *S* is the total number of pairwise nucleotide mismatches, *n* is the number of sequences and *L* is the sequence length. This measure was calculated using the popgenstat.nucleotide_diversity function as implemented in the DendroPy package (Sukumaran & Holder, 2010). To assess whether genetic variation increases with time, sequences were binned per year by registration date of the sample. Then nucleotide diversity was estimated per year and regressed against year of registration. Linear regression was weighted by cohort size per year.

### Distance to vaccine

We tested for changes with time in the distance to vaccine strains by performing linear regression on mismatches in the protein sequence. We expressed distance to the vaccine strain protein sequences as the sum of the substitution scores weighted by the BLOSUM62 scoring matrix. This metric was plotted as a function of the registration date of the sample. Vaccine sequences were removed from the data set to avoid the swamping of a selection signal. This analysis was performed for the entire data set as well as for the two major lineages GI-13 and GI-19.

### N-glycosylation sites

Nucleotide sequences were translated and submitted to the NetNGlyc server 1.0 software for the prediction of N-linked glycosylation sites (Gupta & Brunak, 2002). We adopted the default threshold of 0.5 to indicate potential N-glycosylation sites.

### Accession numbers

The nucleotide sequences analysed in this study were submitted to GenBank and are available under accession numbers OP673683 through OP675435.

## Results

### Quality control

At the start of quality control (QC) there were 2,077 sequences of poultry samples that were submitted to Royal GD between 2011 and 2020. After QC, 1,753 sequences remained (84%). Most of the sequences that failed QC were degenerate, or suspected mixtures.

### Lineage frequencies in time

**Figure 1.**
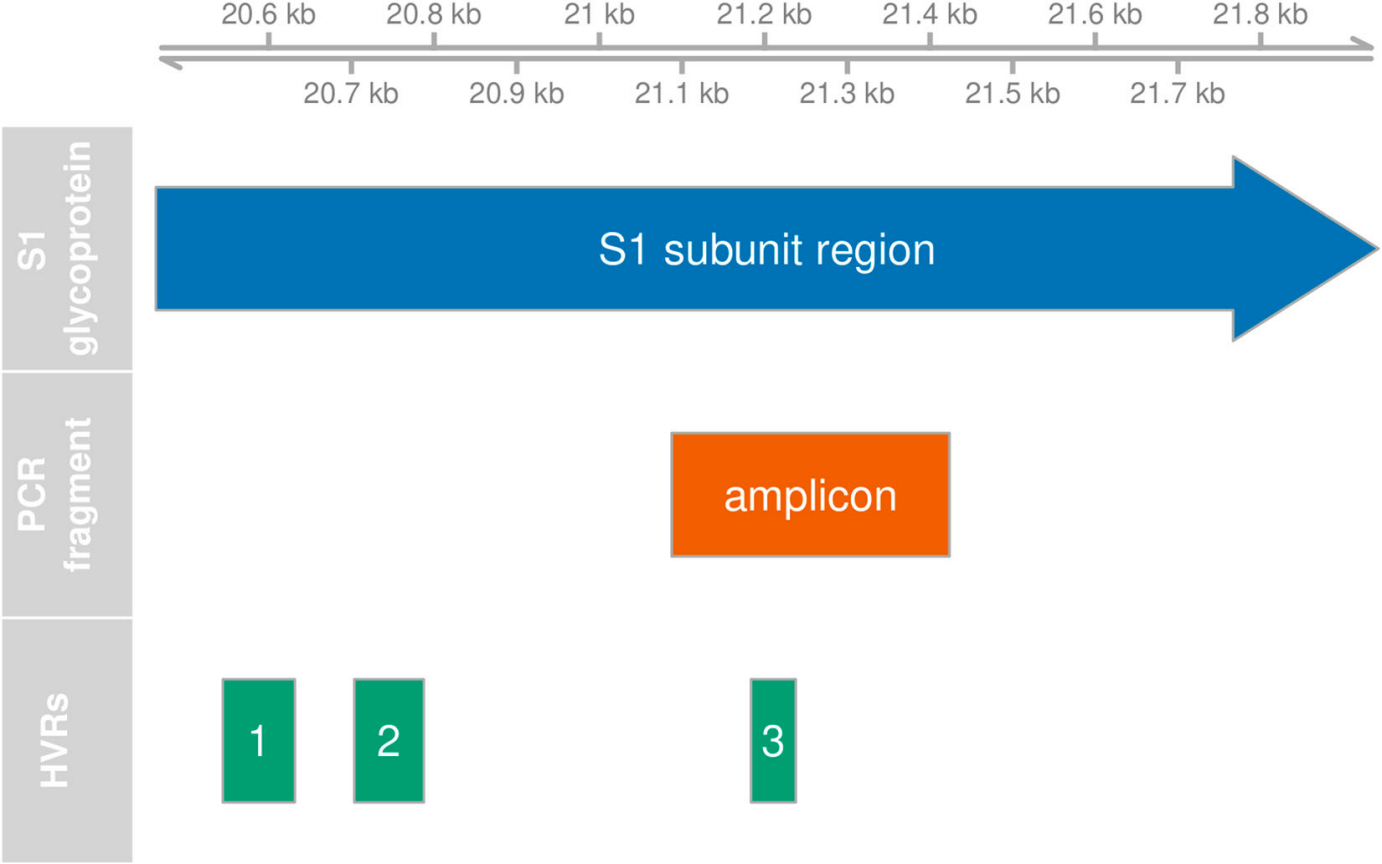
Position of PCR fragment. Genomic feature plot showing the relative positions of the region coding for the S1 glycoprotein, the hypervariable regions (HVR) and the amplified PCR fragment. The scale at the top shows the nucleotide position on the Beaudette reference genome.

**Figure 2.**
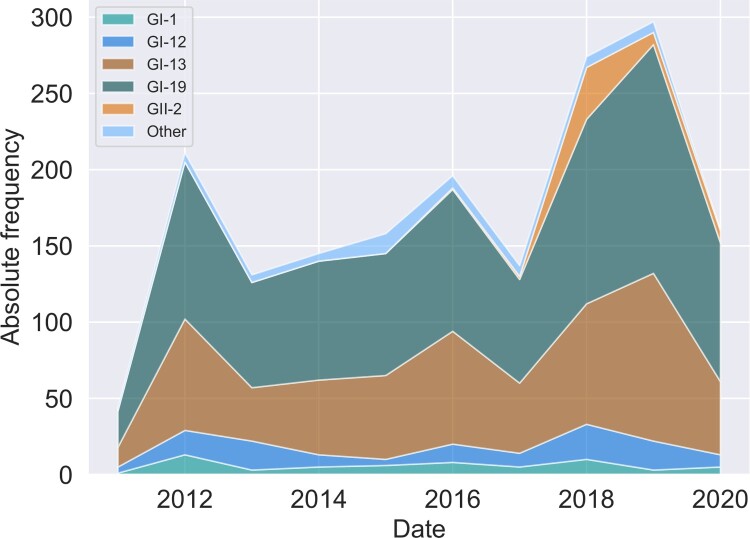
Changes in lineage frequencies. Stacked area chart of the absolute lineage frequencies as a function of time. Lineages were binned per year.

All typings were based on variations in the PCR amplicon which covers residues 241–352 in the C-terminal domain of the translated sequence of the S-gene of the Beaudette reference sequence. This region overlaps HVR3, residues 273-290. The relative position of the amplicon with respect to the S1 subunit gene and its HVRs is shown in Figure 1. Changes in absolute lineage frequencies in time are shown in Figure 2. Two major lineages dominate the graph. Adopting the nomenclature introduced by Valastro *et al*. (2016), we refer to the first as the GI-13 lineage, commonly known as 793B. Several strains within this lineage, like Nobilis 4/91, Gallivac IB88 and Cevac 1/96, were used as parent strains for commercially available vaccines (de Wit, Cook *et al*., 2011). The second major lineage is GI-19, commonly known as QX or D388. We also found appreciable numbers of GI-1 (Mass) and GI-12 (D274). Finally, note the appearance of the GII-2 lineage (D181) from 2017 onwards. Its absence before this date might be an artefact because, although this new lineage would have been detected by genotype-specific RT–PCR for GII-1 strains (also known as D1466), it was not being sequenced until 2017 (Molenaar *et al*., 2020). Since the inclusion of a highly divergent strain halfway through our time window would have severely impacted estimates of divergence and genetic variation with time, 53 GII-2 and nine sequences from other highly divergent lineages were left out of temporal analysis.

To test whether changes in the relative frequencies of the typed lineages are significant, we performed logistic regression with binomial errors on the number of GI-13 and GI-19 sequences with time. Both with and without correction for the GII-2 lineage, the relative frequency of these major strains did not significantly change with time at *P* = 0.05.

### Phylogenetics

**Figure 3.**
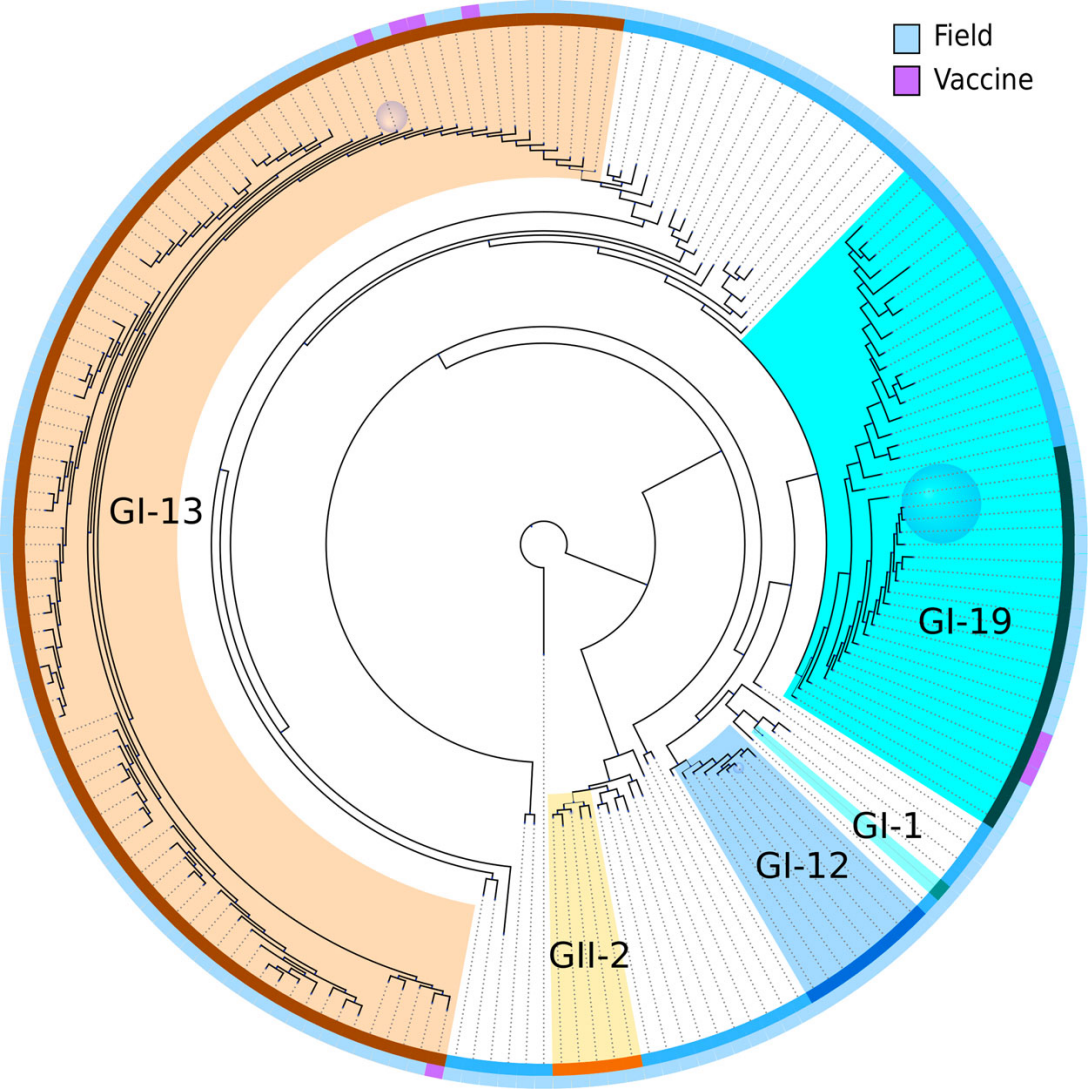
Phylogenetic tree of all partial S1 subunit gene sequences. The tree has been rooted by setting a known basal unique variant as outgroup. Clades where the terminal nodes had less than 0.01 subsitutions per site distance on average were collapsed. Sizes of the blue sphere faces are proportional to the number of sequences that have been collapsed into the associated clades. The outer rim shows vaccine status and the inner rim shows typed lineage. Lineage colour code matches that of Figure 2. Coloured branches indicate descendants of the most recent common ancestor of lineages indicated on the inner rim. The lineage type and vaccine status of collapsed nodes were determined by majority vote.

A phylogenetic tree was built using IQ-TREE (Figure 3). Removal of recombinants and mixtures during QC allowed proper separation of the GI-13 and GI-19 lineages. With the exception of GI-19, typed lineages were assembled into monophyletic clades. The branch leading to GII-2 and the single basal sequence collects all sequences outside of the GI genotype present in our dataset. The basalmost sequence is a unique variant outside of the GI and GII lineages, which was used as an outgroup to root the tree.

### Vaccine sequences and outbreaks

**Figure 4.**
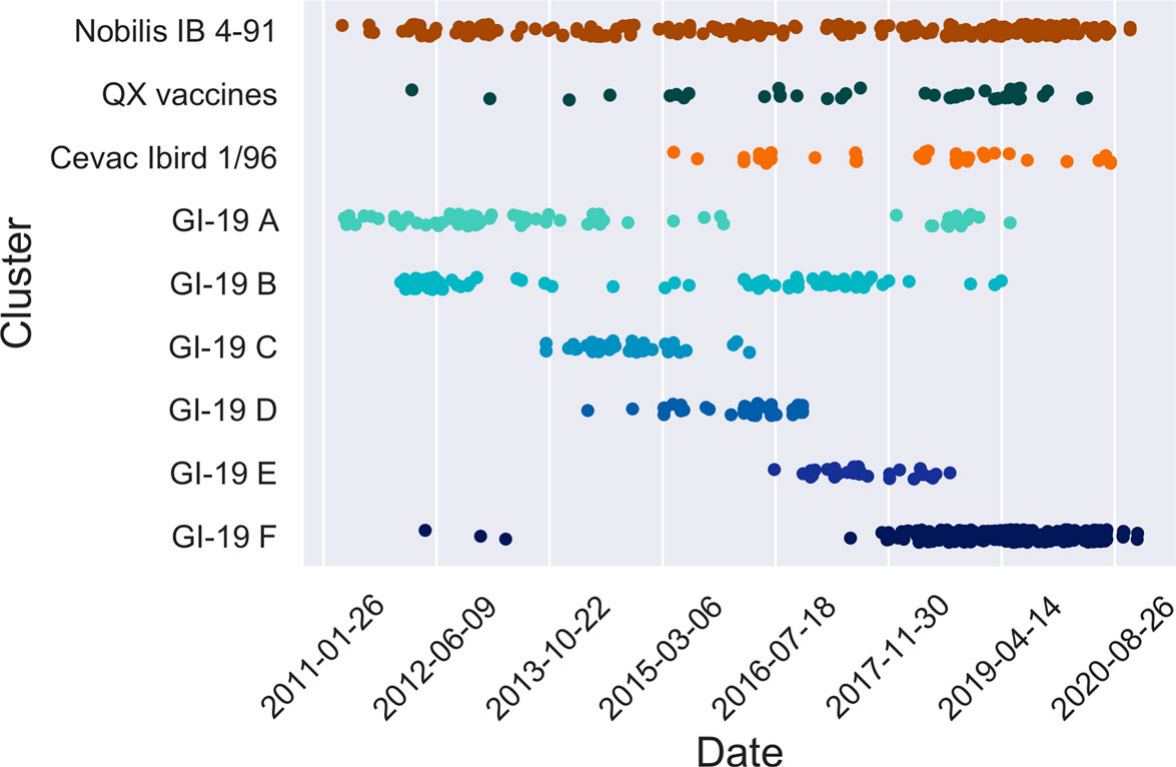
Succession of sequence clusters. Appearance of clusters of similar sequences in broiler chickens in time. The upper three clusters are known vaccines, the lower six are non-vaccine clusters. Note the succession in time of the latter. QX vaccines collects sequences similar to Nobilis IB Primo QX and Poulvac IB QX. Nobilis IB 4-91 was already marketed prior to 2010, Poulvac IB QX was introduced around 2010-2011, Nobilis IB Primo QX and Cevac Ibird (1/96) vaccine were first introduced around 2013-2014.

A conspicuous feature of the data set was the presence of large clusters of near identical sequences. Some of these corresponded to vaccines, which have remained unchanged and were administered at a large scale. To identify potential vaccines in our data set, we screened the data set for large clusters of highly similar sequences (at least 20 sequences with at most four nucleotide mismatches). Then we tested for overrepresentation of samples from broilers within those clusters, since broilers are expected to be predominantly infected by lingering vaccine strains due to their short life span and recent use of these live vaccines. This resulted in two clusters in the GI-13 lineage and seven clusters in the GI-19 lineage. We screened for five commercially available vaccines in GI-13 and GI-19 known to be used in the Netherlands (Table 1) and found 660 vaccine sequences (38%). The two clusters in GI-13 indeed matched the Nobilis IB 4-91 and Cevac Ibird vaccine sequences (Table 2, Figure 3). Of seven clusters in the GI-19 lineage, only one corresponded to two highly similar known vaccines (Nobilis IB Primo QX and Poulvac IB QX). The position of the vaccine sequences is indicated in the phylogenetic tree (Figure 3). All sequences assigned to Nobilis IB 4-91 were located on the top left part of Figure 3, whereas Cevac Ibird vaccine sequences appeared in the lower left part. The group of vaccine sequences in the GI-19 lineage corresponds to the cluster of combined QX vaccine sequences. The remaining six clusters within lineage GI-19 were all collapsed into a blue sphere to prevent Figure 3 from cluttering up. To investigate these six clusters, we plotted their registration dates and found them to have appeared in short single bursts of about a year (clusters C, D, and E), 3 years (F) or a longstanding burst of 6–7 years with a varying number of detections in time (A) (Figure 4). It is noteworthy that broilers were significantly overrepresented in samples typed for GI-19 (60% against 44% overall) whereas broilers were significantly underrepresented in samples typed for GI-13 (30%).

**Table 1.** Number of vaccine sequences.

Vaccine	Lineage	Times typed
Nobilis IB Primo QX	GI-19	69
Poulvac IB QX	GI-19	14
Nobilis IB 4-91	GI-13	532
Cevac Ibird 1/96	GI-13	39
Gallivac IB88	GI-13	6

Number of typings at 95% sequence identity of several known commercially available vaccines in our data set.

**Table 2.** Clusters of similar sequences in broilers.

Cluster^a^	Sequences^b^	Unique sequences^c^	Dominant sequence count^d^	aa differences QX vaccine^e^
GI-19 A	80	22	54	T274S
GI-19 B	91	12	76	T274S
GI-19 C	39	8	27	S248N, T274S, S318T
GI-19 D	41	4	35	S248N, T274S, N288S, S318T
GI-19 E	30	8	20	S248N, T274S, N288S
GI-19 F	267	47	184	T274S, N288S
Nobilis IB Primo QX + Poulvac IB QX	46	5	32	0
Cevac Ibird 1/96	37	6	30	NA^f^
Nobilis IB 4-91	245	36	202	NA

Three of the clusters correspond to known vaccines, the others are probably epidemics.

^a^ Either cluster name, or the vaccine it corresponds to.

^b^ Number of sequences in the cluster.

^c^ Number of unique sequences in the cluster.

^d^ Count of the most abundant haplotype.

^e^ Protein sequence differences with QX vaccines (QX strains only).

^f^ NA = Not applicable.

### Episodic selection

**Figure 5.**
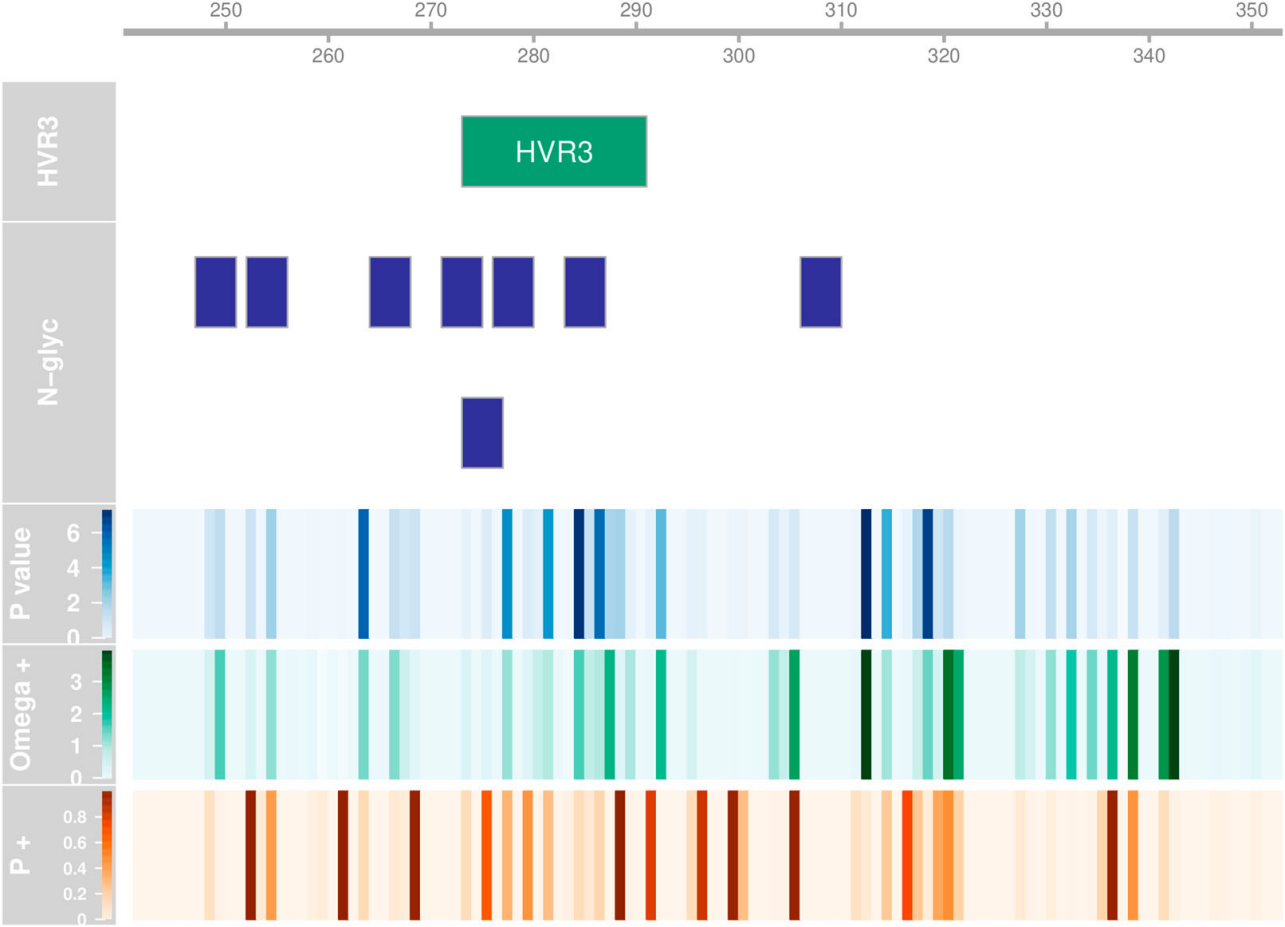
Diversifying selection per codon. Hyphy MEME metrics indicating the strength and distribution of diversifying selection for every codon position. The scale at the top shows the residue position for the S-protein of the Beaudette reference. The relative position of HVR3 is also indicated, as well as the positions of predicted N-glycosylation sites. The upper heat map panel shows minus log_10_ transformed *P*-value for episodic diversification. The middle panel shows the log_10_ transformed strength of diversifying selection (ω+) and the lower panel shows the proportion of branches under diversifying selection (p+).

In order to assess whether positive selection was operating on our fragment of the S1 gene, we submitted our complete alignment to MEME, which screens for episodic increases in the rate of nonsynonymous substitutions over synonymous substitutions in a phylogenetic tree (Murrell *et al*. 2012). MEME reported episodic diversifying positive selection at 28 sites (24%) at *P* < 0.1 (Table 3, Figure 5). To check for the robustness of findings, we corrected for multiple comparisons by controlling the false discovery rate at 5% and found 19 sites to remain significant at this level.

**Table 3.** Sites under diversifying selection.

Site^a^	*P* value^b^	α^c^	ω^−d^	p^−e^	ω^+f^	p^+g^	Significant GI-19^h^	Significant GI-13^i^
248	0.004	2.49	0.14	0.906	6.93	0.094	No	Yes
249	0.008	0.24	1.00	0.937	36.16	0.063	No	No
254	0.005	0.10	0.48	0.487	17.68	0.513	Yes	Yes
263	< 0.001	0.26	0.00	0.806	28.16	0.194	No	Yes
266	0.022	0.28	0.00	0.896	23.82	0.104	Yes	No
267	0.075	1.19	0.00	0.928	6.37	0.072	Yes	No
275	0.069	0.96	0.00	0.526	3.30	0.474	No	No
277	< 0.001	0.31	0.00	0.688	18.91	0.312	Yes	Yes
279	0.071	1.60	0.17	0.504	3.13	0.496	Yes	No
281	< 0.001	0.76	1.00	0.832	16.09	0.168	Yes	Yes
284	< 0.001	0.45	1.00	0.863	47.59	0.137	Yes	Yes
285	0.046	0.44	0.31	0.968	33.12	0.032	No	No
286	< 0.001	1.08	0.19	0.708	6.94	0.292	No	Yes
287	0.025	0.85	0.16	0.998	162.94	0.002	Yes	No
292	< 0.001	0.63	0.49	0.996	176.33	0.004	Yes	No
305	0.077	0.00	0.00	0.936	5471.48	0.064	No	No
312	< 0.001	0.37	0.57	0.981	144.72	0.019	Yes	No
314	< 0.001	0.25	1.00	0.784	22.98	0.216	No	Yes
316	0.029	1.49	0.00	0.499	3.28	0.501	Yes	No
317	0.004	0.82	0.21	0.698	6.46	0.302	Yes	Yes
318	< 0.001	0.71	0.13	0.953	41.82	0.047	No	Yes
319	0.008	0.62	0.20	0.634	8.66	0.366	No	No
320	0.012	0.00	0.00	0.170	1467.26	0.830	No	Yes
327	0.006	0.62	0.00	0.910	11.08	0.090	No	Yes
330	0.073	1.03	0.55	0.968	15.55	0.032	No	No
332	0.008	0.24	1.00	0.992	65.47	0.008	No	Yes
338	0.014	0.00	0.00	0.593	2205.74	0.407	No	No
342	0.012	0.00	0.00	0.973	9344.90	0.027	Yes	No

Sites reported to be under diversifying selection by HyPhy MEME.

^a^ Codon reference position.

^b^ Asymptotic *P* value for episodic diversification.

^c^ Synonymous substitution rate.

^d^ Strength of purifying selection (β^−^/α).

^e^ Proportion of branches under purifying selection.

^f^ Strength of diversifying selection (β^+^/α).

^g^ Proportion of branches under diversifying selection.

^h^ Significant positive selection detected within the GI-19 clade at *P* = < 0.1.

^i^ Significant positive selection detected within the GI-13 clade at *P* = < 0.1.

### Genetic variation in time

**Figure 6.**
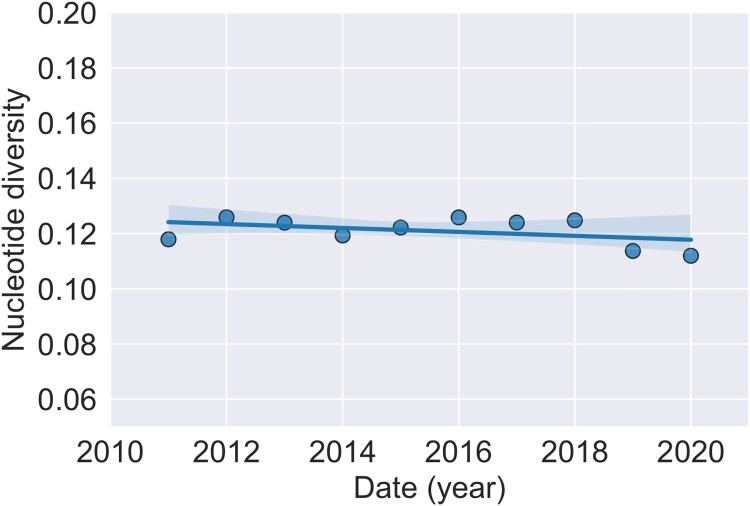
Change in nucleotide diversity. Nucleotide diversity as a function of time (year). Least-square regression line with 95% confidence limits is also plotted.

The analysis on changes in genetic variation with time was performed on the data set excluding all non-GI sequences. There were 261 segregating sites in the total 336 nucleotide alignment. The overall nucleotide diversity *π* was found to be 0.122. One prediction of diversifying selection operating on the population is that the genetic variation would increase with time. This was assessed by performing a linear regression of nucleotide diversity per year, with time as the independent variable (Figure 6). However, no significant change in overall nucleotide diversity with time was found in this time period (*F_1,8_* = 4.3, *P* = 0.07 n.s.).

### Distance from vaccine

To assess whether immune escape from vaccine-induced immunity is driving selection in the S1 HVR3 fragment, we plotted distance from the vaccine sequences as a function of time (Online Supplementary Figure 2(a,b)). We focused on distance from the two most abundant vaccine sequences in our data set. Regardless of whether divergence occurs stepwise or gradually, increasing divergence should result in positive slopes. However, we could not detect any increase in protein distance for either vaccine as the slope of regression was non-significant for Nobilis IB 4-91 (*F_1,1361_* = 0.4, *P* = 0.55 n.s.) and even decreased for Nobilis IB Primo QX / Poulvac IB QX (*F_1,1361_* = 7.8, *P* = 0.005). Also when we considered only selected sites and/or considered only the vaccine-homologous lineage, i.e. GI-13, or GI-19, we found no significant increase in distance to vaccine (data not shown). Hence, no overall divergence from vaccine sequences was apparent from this analysis.

### N-glycosylation sites

Glycosylation of the S1 protein has been suggested to be relevant for viral receptor binding and viral entry into the cell (Zheng *et al*., 2018) and shields the viral protein from neutralizing antibodies as described for influenza (Wei *et al*., 2010). To investigate this aspect of our data set, we used the NetNGlyc server to predict the presence and absence of N-linked glycosylation sites. In our fragment, we found 11 candidate asparagine positions with support from at least one sequence. Three of those predicted sites occurred in only one or two sequences so were dropped from the analysis. The positions of the remaining eight predicted glycosylation sites are shown in Figure 5. The frequency of these glycosylation sites ranged from 0.5 to 98.4% of all sequences in our data set.

We investigated whether some of the sites experiencing episodic diversifying positive selection coincided with the Asn-Xaa-Ser/Thr glycosylation motifs of the predicted glycosylation sites. This was the case for the codons at residues 248, 249, 254, 266, 275, 277, 284 and 285. One of those residues (248) was polymorphic among the GI-19 clusters we found to appear in successive bursts (Table 2). Another interesting question is which glycosylation sites were predicted to be present in vaccine stocks. The sequences associated with the four most abundant vaccines in our data set (Cevac Ibird, Nobilis IB 4-91, Nobilis IB Primo QX and Poulvac IB QX) all have predicted glycosylation sites at positions N247, N271, N276 and N306. Remarkably, the two QX vaccines are lacking the predicted site at position N264 present in most GI sequences, including the two other vaccines. Finally, we studied the phylogenetic distribution of the predicted glycosylation sites (Online Supplementary Figure 3). We found that glycosylation of positions N247 and N264 was predicted to be restricted to GI, whereas glycosylation at position N252 was predicted to be limited to GII. Predicted glycosylation at position N283 was found to be specific to the GI-1 lineage. The distribution of glycosylation at other positions did not follow a clear pattern.

## Discussion

In this study we examined a data set of 1753 partial S1 gene sequences encompassing HVR3 with the aim to describe within and between lineage dynamics and explore several factors that may affect such dynamics.

We observed two dominant lineages in our data set i.e. GI-13 and GI-19. This superficially resembles the situation observed for OC43 human coronavirus where, based on the phylogeny of the spike protein, two lineages can be distinguished that are believed to be engaged in epidemic switching (Kistler & Bedford, 2021). In contrast, in our study, the relative proportions of both lineages remained stable with time. At the within-lineage level, we found an ongoing succession of outbreaks of related GI-19 strains. Since these clusters only appear during specific time windows, we infer that these clusters are not vaccine related but most likely represent historical outbreaks. This type of epidemic switching between genotypes within lineages has also been described in other viruses, e.g. in human coronavirus OC43 (Komabayashi *et al*., 2021). This phenomenon could reflect the continuous competitive displacement of variants by ongoing adaptive evolution. Alternatively, it might result from a succession of distinct introductions and extinctions from field strains into livestock. Interestingly, broilers are strongly overrepresented in samples typed for GI-19, and GI-19 infections appeared in a succession of large outbreaks. On the other hand, broilers are underrepresented in samples typed for GI-13, with GI-13 sequences being highly diverse and predominantly appearing as incidental infections of layers. It is tempting to speculate that this pattern originates in genetic differences between lineages in the ability to exploit different risk factors between the two farming types (e.g. life span, vaccination programmes). Any future study on the selective pressures acting on IBV would likely benefit from stratifying data obtained from broilers and layers/breeders.

We detected signals of selection in the S1 gene fragment, but it remains difficult to elucidate what factors drive these changes. In principle, this signal could derive from adaptive evolution to counteract host immunity at a select few residues. Important clues derive from functional descriptions of the encoded protein. The sugar-binding domain of the S1 spike protein has been shown to be located in the N-terminal domain of the spike (Promkuntod *et al*., 2014). The fragment we have analysed covers residues 241–352 in the C-terminal domain and overlaps the HVR3 sequence. The C-terminal domain includes two extended loops which function as putative receptor-binding motifs (RBMs). The targeted receptor is as yet unknown (Shang *et al*., 2018). Zheng *et al*. (2018) published a functional study of N-linked glycosylation in the S-gene of IBV. In the region considered in this study, they reported five putative glycosylation sites. Based on studies of cell–cell fusion and virus recovery upon mutation, they found positions N276 and N283 to be of critical importance, although the function of N283 was found to be independent of glycosylation. This result is concordant with our findings, where the motifs associated with both sites overlap codons experiencing episodic diversifying positive selection. This emphasizes the need to complement sequence analysis with functional studies. Better knowledge of the antigenic structure of the spike would indicate relevant positions for immune escape and could substantially strengthen conclusions.

There is support for the hypothesis that live attenuated vaccine usage can impact IBV genetic population structure (Jackwood and Lee, 2017). In our study, despite evidence for episodic selection acting on multiple sites in the fragment, we could not link the mutations to escape from vaccine-induced immunity. The frequency of vaccine-homologous lineages did not decrease, no increase in genetic variation with time was detected, and the sequences did not grow more divergent from vaccine sequences in the examined time window. Hence, we were unable to demonstrate that IBV is adapting to the selection pressure incurred from vaccination. Neither are we confident to conclude that no adaptation occurs, since it has been suggested that only a few amino acid differences in the S1 protein suffice to escape vaccine-induced protection and those might be hard to detect in our study (Abdel-Moneim *et al*., 2006). Such cases have been demonstrated for influenza A, where only five antigenic sites were sufficient to account for antigenic drift (Koel *et al*., 2014). In addition, mutations occurring outside of the sequenced fragment remained invisible to us. As we cannot exclude that immune-driven natural selection occurs in other spike regions that contain antigenic sites, e.g. the more N-terminal S1 domain that contains the sialic acid binding site, the lack of divergence in our data set does not conclusively establish that field viruses are not adapting to vaccines. We did demonstrate that several sites experiencing episodic selection coincide with N-glycosylation motifs. These present useful candidates for functional studies. Better knowledge of the antigenic structure of the spike protein may help elucidate the selective factors driving sequence evolution.

This study is based on a unique data set. It comprises a relatively large and homogeneous database, covers a long period and is associated with a rich body of metadata. Also, it enables us to investigate the evolution of IBV experiencing massive vaccination in a large and dense population. On the other hand, we only had access to a partial sequence of the S1 gene. In future studies, full spike sequences or full genomes should be analysed. In addition, our analysis may have benefited from including detailed information, such as which vaccines were used, when vaccination was applied and the age of the flock. Since the IBV sequences were collected for a different purpose than this study, these data were not available to us. Finally, during QC many samples were discarded because of being degenerate or suspected mixtures. This is a consequence of the application of live vaccines that may persist and result in multiple infections. Separation of mixtures of strains is non-trivial and will continue to plague sequencing efforts.

Our results provide several interesting insights into within- and between-lineage dynamics of IBV in a long-term vaccinated poultry population. Lineage frequencies appeared to remain stable, despite ongoing episodic selection at the spike protein and succession of variants. We have also shown indications that certain lineages preferentially occur in either broiler, or layer production types.

## Ethical statement

The study reported in this paper was non-interventional and only used existing sequence data from samples that were submitted to Royal GD for routine diagnostic purposes.

## Supplementary Material

Supplemental Material

Supplemental Material
