# Supplementary material for: Genetic analysis of infectious bronchitis virus (IBV) in vaccinated poultry populations over a period of 10 years
Source: Avian Pathol. 2023 Feb 24;52(3):157–67. doi: 10.1080/03079457.2023.2177140 (PMC10243407; doi:10.1080/03079457.2023.2177140)

Supplementary Figure 1: Overview of the QC-procedure


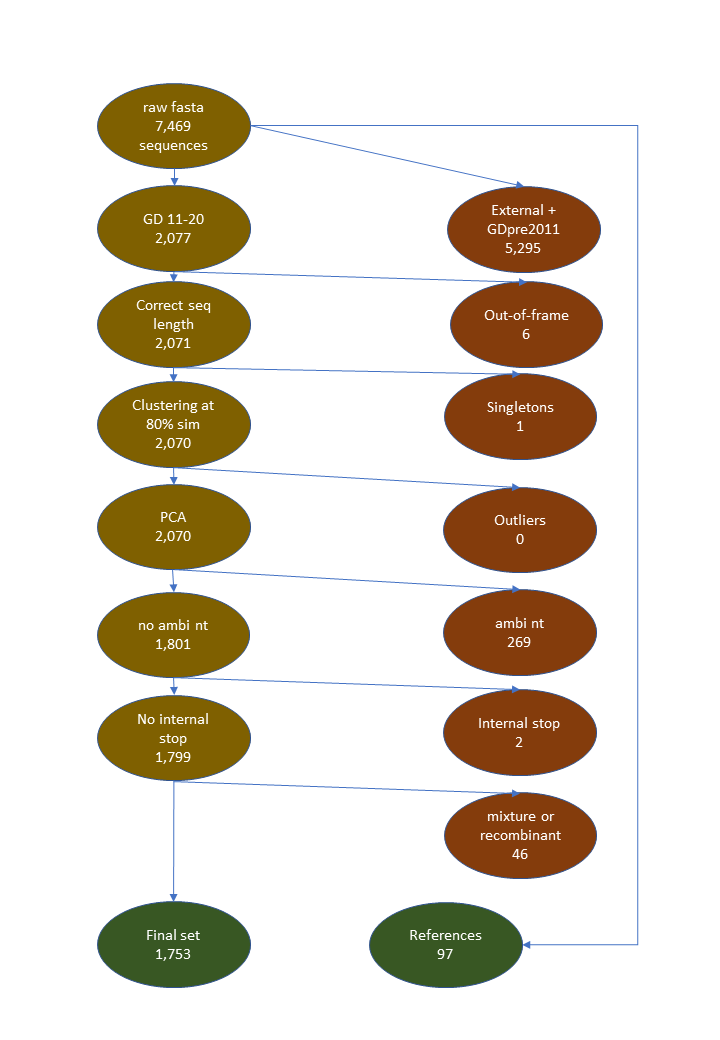


Supplementary Figure 2: Protein distance from a) Intervet Nobilis 4-91 vaccine and b) IB Primo QX vaccine sequences with time. Least-square regression lines with 95% confidence limits are plotted as well. Lineage color code matches that of Figure 2.


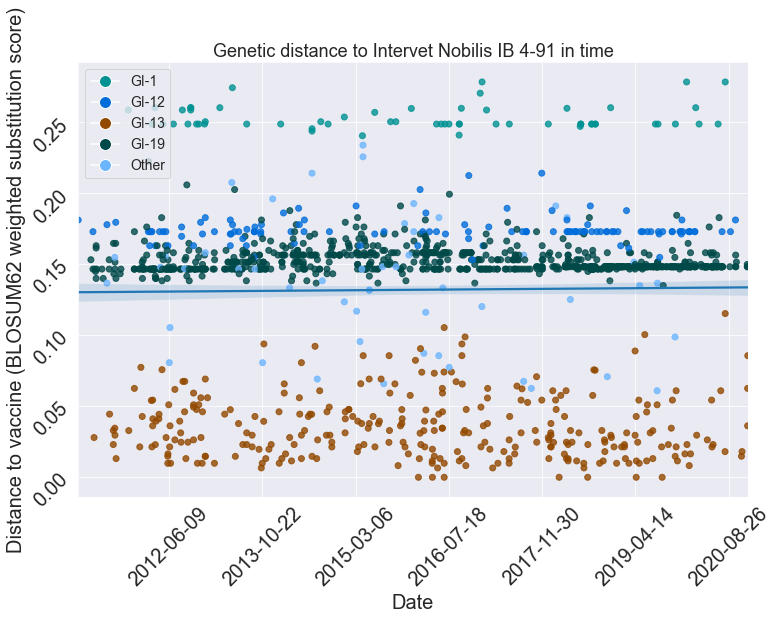


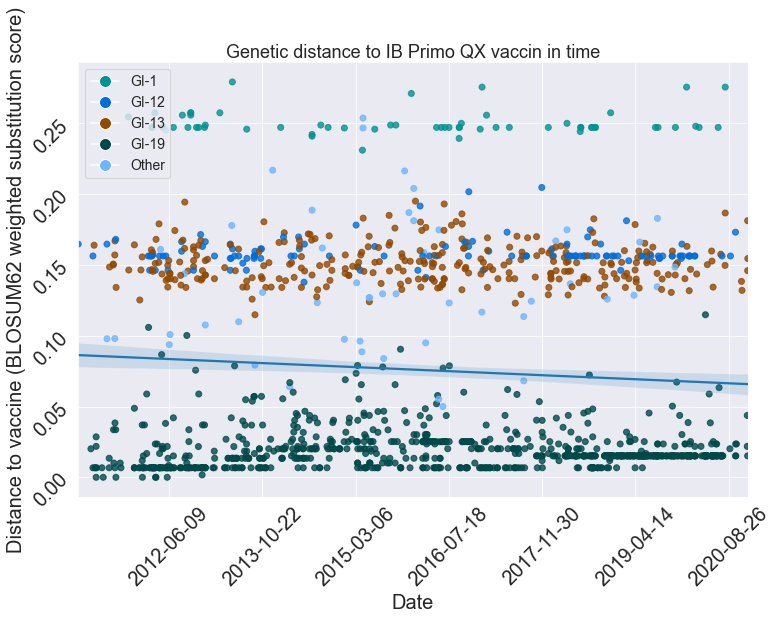


Supplementary Figure 3: Distribution of eight predicted N-glycosylation sites in the phylogenetic tree based on partial S1 subunit gene sequences. The outer rim shows presence (purple) or absence (yellow) of predicted N-glycosylation sites at a given position. The tree has been rooted by setting a known basal unique variant as outgroup. Clades where the terminal nodes had less than 0.01 subsitutions per site distance on average were collapsed. Size of the blue sphere faces are proportional to the number of sequences that have been collapsed into the associated clades. The inner rim shows typed lineage. Coloured branches indicate descendants of the most recent common ancestor of lineages indicated on the inner rim. The lineage type and glycosylation site status of collapsed nodes was determined by majority vote.


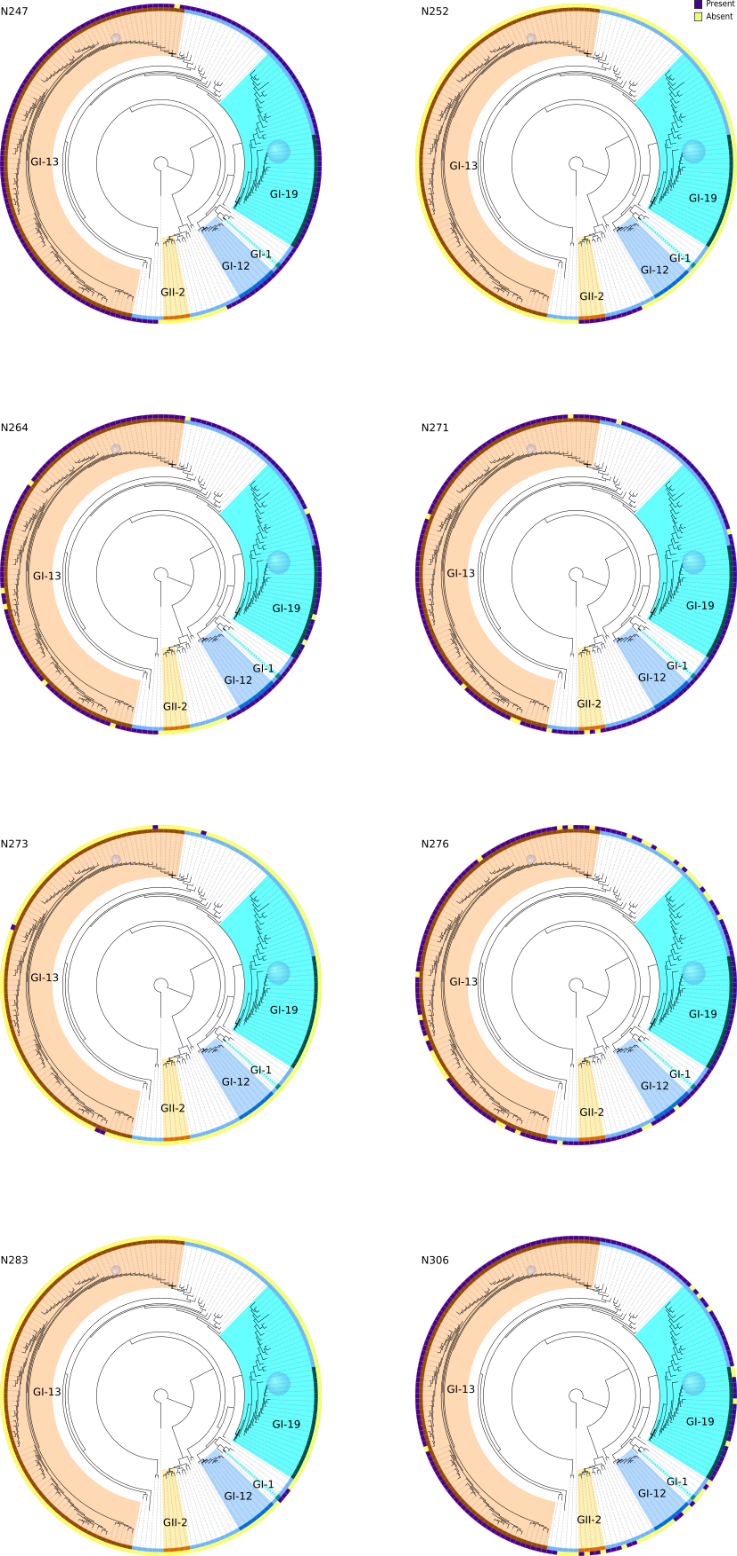

Supplement: Supplemental Material [file CAVP_A_2177140_SM4980.docx]
